# The Effect of Pooling on the Detection of the Nucleocapsid Protein of SARS-CoV-2 with Rapid Antigen Tests

**DOI:** 10.3390/diagnostics11071290

**Published:** 2021-07-19

**Authors:** Tim Berking, Sabrina G. Lorenz, Alexander B. Ulrich, Joachim Greiner, Eric Kervio, Jennifer Bremer, Christina Wege, Tatjana Kleinow, Clemens Richert

**Affiliations:** 1 Institute of Organic Chemistry, University of Stuttgart, Pfaffenwaldring 55, 70569 Stuttgart, Germany; tim.berking@oc.uni-stuttgart.de (T.B.); sabrina.lorenz@oc.uni-stuttgart.de (S.G.L.); alexander-bernhard.ulrich@oc.uni-stuttgart.de (A.B.U.); eric.kervio@oc.uni-stuttgart.de (E.K.); jennifer.bremer@oc.uni-stuttgart.de (J.B.); 2 Institute of Aircraft Design, University of Stuttgart, Pfaffenwaldring 31, 70569 Stuttgart, Germany; Greiner@ifb.uni-stuttgart.de; 3 Institute of Biomaterials and Biomolecular Systems, University of Stuttgart, Pfaffenwaldring 57, 70569 Stuttgart, Germany; christina.wege@bio.uni-stuttgart.de (C.W.); tatjana.kleinow@bio.uni-stuttgart.de (T.K.)

**Keywords:** SARS-CoV-2, rapid antigene test, pooling, nucleocapsid protein, lateral flow test

## Abstract

The COVID-19 pandemic puts significant stress on the viral testing capabilities of many countries. Rapid point-of-care (PoC) antigen tests are valuable tools but implementing frequent large scale testing is costly. We have developed an inexpensive device for pooling swabs, extracting specimens, and detecting viral antigens with a commercial lateral flow test for the nucleocapsid protein of SARS-CoV-2 as antigen. The holder of the device can be produced locally through 3D printing. The extraction and the elution can be performed with the entire set-up encapsulated in a transparent bag, minimizing the risk of infection for the operator. With 0.35 mL extraction buffer and six swabs, including a positive control swab, 43 ± 6% (*n* = 8) of the signal for an individual extraction of a positive control standard was obtained. Image analysis still showed a signal-to-noise ratio of approximately 2:1 at 32-fold dilution of the extract from a single positive control swab. The relative signal from the test line versus the control line was found to scale linearly upon dilution (R^2^ = 0.98), indicating that other pooling regimes are conceivable. A pilot project involving 14 participants and 18 pooled tests in a laboratory course at our university did not give any false positives, and an individual case study confirmed the ability to detect a SARS-CoV-2 infection with five-fold or six-fold pooling, including one swab from a PCR-confirmed COVID patient. These findings suggest that pooling can make frequent testing more affordable for schools, universities, and similar institutions, without decreasing sensitivity to an unacceptable level.

## 1. Introduction

Identifying asymptomatic spreaders of viral infections is an important task in a pandemic. This is particularly true for COVID-19, a viral disease with many ‘silent spreaders’ that is proving difficult to control within a population [1,2]. Frequent testing of a large portion of the population is expected to reduce the spread of the disease, because spreading events can be avoided. Monitoring is most effective when testing is performed frequently, and when an approach is employed that provides results quickly after collecting the specimens, even if the method is less sensitive than RT-PCR [3]. Lateral flow assays (LFAs) that detect a viral antigen are among the rapid tests that are attractive in this context [4]. They produce results within 15–30 min, without the need for a laboratory, and can show high specificity [5]. Several studies have validated lateral flow rapid antigen tests in the current pandemic, focusing on the comparison between different test systems, validation in non-clinical settings, or correlation with infectivity in vitro [6,7,8,9,10].

Even though lateral flow antigen tests can be mass-produced, the cost of mass testing individuals on a regular basis can become prohibitive, even for developed countries [2,11]. Pooling of samples that are then analyzed in a single procedure is known to lower the cost of testing in scenarios with low incidence [12]. Further, pooled testing reduces the effort for participants, making it easier to monitor over extended periods of time. Pooling has been reported for PCR tests detecting SARS-CoV-2 [13,14,15,16]. A recent report from a company suggests that pooled samples can successfully be analyzed by lateral flow point of care (PoC) test cassettes [17], but pooling has not become an established practice for rapid antigen tests. One reason for this may be the need for biosafety measures and laboratory equipment to perform the pooling steps, which counterbalances the ease of use for LFAs at the point of need. Such complications may be overcome by a device suitable for the pooling, extraction and analysis of specimens. Here we report such a device, together with results from pooling experiments in a laboratory setting, a pilot project in a university course, and a case study involving swabs from a PCR-confirmed COVID-19 patient.

## 2. Materials and Methods

Components of the Panbio™ COVID-19 Ag Rapid Test Device were used (Abbott Rapid Diagnostics Jena GmbH, Jena, Germany), for which the manufacturer reports a sensitivity of 91.4% (nasopharyngeal swab versus nasopharyngeal PCR) and a specificity of 99.8% (https://www.globalpointofcare.abbott/en/product-details/panbio-covid-19-ag-antigen-test.html, accessed on 16 July 2021). Alternatively, the SARS-CoV-2 rapid antigen test, manufactured by SD Biosensors (Suwon-si, Gyeonggi-do, Korea) and distributed by Roche Diagnostics GmbH (Mannheim, Germany) was used. Both the Abbott and the Roche tests detect the nucleocapsid protein of SARS-CoV-2 as antigen and contain a control line detecting chicken IgY. The laboratory experiments were performed with the nasopharyngeal version of the test, whereas the pilot project and case study used the nasal version. The supplier of the tests confirmed that the two versions of the test are identical, except for the swabs. According to the manual, the extraction buffer of the test kit contains tricine as buffer component, NaCl, Tween 20 as non-ionic surfactant, sodium azide as bacteriostatic (listed as <0.1%), and Proclin 300 as other preservatives/biocides. Dilution experiments with water used Kabi Ampuwa sterile water (Fresenius Kabi Deutschland GmbH, Bad Homberg, Germany) for injection purposes. Positive control swabs provided with the Abbott kits were used as a source of SARS-CoV-2 antigen. Either the Noble Biosciences NFS-1 nasopharyngeal swabs supplied with the Abbott test kit (nasopharyngeal version) were used (‘50 µL swabs’) or singly wrapped, sterile, cotton wool swabs (5 mm head) with a wooden stem (150 mm length) from neoLab (Heidelberg, Germany) as larger specimen alternatives (‘100 µL swabs’). For laboratory experiments, nasopharyngeal or oropharyngeal swab specimens were self-collected from healthy volunteers among the authors who had tested negative in professional tests performed by physicians, using the Roche test. Specimens for the pilot project and the case study were collected by first wetting the swabs with saliva in the buccal cavity for 30 s, followed by oropharyngeal and then nasopharyngeal swabbing, as typically performed when collecting specimens for PCR analysis, and were analyzed using the Abbott PanBio test cassettes. Extraction buffer was the unmodified buffer from the Abbott PanBio test. Plastic bags were zip lock all-purpose/freeze bags made of polyethylene suitable for transporting liquids in carry-on luggage in the EU, holding either up to 1 L volume or up to 3 L volume (both from QuickPack, Renningen, Germany). Syringes were 10 mL Amefa LUER single-use sterile syringes (B. Braun, Melsungen, Germany). The holder for the syringe body acting as pooling container and the lateral flow test cassette was produced by 3D printing on a Prusa MK2S printer with PLA (polylactide) as material, instructed by CAD data in STL format. All photographs of the laboratory study were taken with the camera of a Samsung Galaxy S20+ (SM-G985F) cellular phone, whereas images of the pilot project study and the case study were a variety of different smartphones. All image analysis was performed using the free program ImageJ (NIH). Details of the image analysis are provided in the Supplemental Material.

## 3. Results

**Pooling Set-up.** We defined the following criteria for the test method to be developed: The method had to use inexpensive materials, including sterile, singly wrapped swabs and a sterile mixing container. The analytical procedure had to be based on a commercial, well established LFA system, and all steps had to be feasible without additional biochemical components and without the need for laboratory equipment. These criteria led to the system described here.

**Figure 1:**
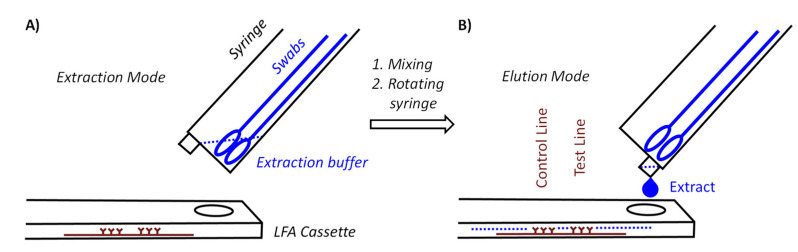
Principle of pooling and extraction in a syringe acting as a container and elution onto a lateral flow assay (LFA) cassette for analysis. (**A**) Container in extraction mode; thorough mixing is induced by rotating the bundle and moving individual swabs. (**B**) Elution, induced by rotating the syringe by 180° to allow for the extract to drip onto specimen well of the LFA cassette.

Figure 1 shows two core components of our device. One of those components is a syringe for medical use that functions as a container for the swabs and as vessel in which the mixing occurs. With the goal to achieve pooling for at least five specimens, we tested several syringe sizes and swabs and settled on 10 milliliter (mL) syringes with the central plunger removed as containers. They will hold approximately ten flocked nylon swabs of the type included in both the Abbott PanBio and the Roche rapid antigen tests or six conventional sterile flocked cotton swabs with wooden stem, without losing the mobility necessary for thorough mixing. The latter swabs cost a fraction of the nylon swabs, hold twice as much specimen, and are more pleasant when used in the mouth.

**Figure 2:**
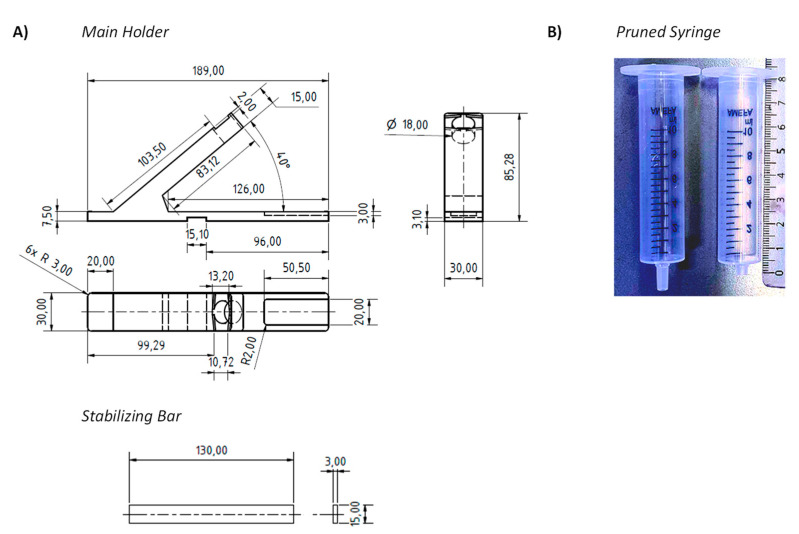
Components of the device. (**A**) Technical drawings showing the dimensions (in mm) of the main holder, the stabilizing bar of the holder, and (**B**) photograph of an unmodified 10 mL syringe next to a syringe used as pooling and extraction container that had its LUER fit pruned to reduce void volume. The length ruler next to the pruned syringe shows the length in cm.

Figure 2 shows the holder of the device and the final form of the syringe, as employed in our current procedure. The holder shown in Figure 2A has an arm with a circular opening for the syringe and indentations next to this opening that arrest the syringe in extraction or elution mode. The base of the holder has another indentation, where the LFA cassette is held in place. Further, there is a small indentation at the center bottom of the base plate, into which a stabilizing bar is pushed to provide mechanical stability to the assembly and to prevent it from tipping over during the extraction and elution steps. While initial prototypes were made of solid PVC, both the main holder and the stabilizing bar are now produced by us by 3D printing in an inexpensive printer. In our currently preferred method for testing, the holder is disinfected and recycled when the test outcome is negative but is disposed of when a test is positive. Our cost of the material for printing both holder and stabilizing bar is currently 1.54 €, so that the method is inexpensive, even if the holder is treated as a disposable item in each test with swab pooling.

**Figure 3:**
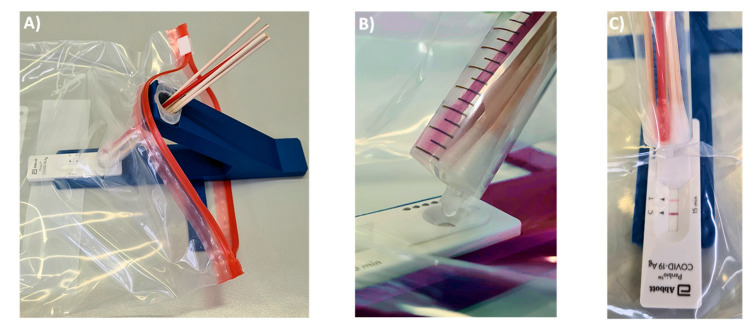
Photographs of testing with the device in a polyethylene bag with zip lock for encapsulation after loading. (**A**) Device loaded with five 100 µL flocked cotton swabs holding specimen and one positive control swab (red). (**B**) Close-up showing elution of the extract after thoroughly mixing for 3 min. Three drops are required to elute 100 µL from the pruned syringe, corresponding to 5 drops from the original Abbott extraction container. (**C**) Result of a test, viewed through the encapsulating bag after 15 min.

One modification was made to the commercial 10 mL syringes used as container. Their LUER fit tip was pruned to avoid retention of a significant fraction of the extract in the form of the void volume of the device. Fully removing the tip led to uncontrolled elution and very large drops. Leaving the LUER opening unchanged retained too much of the valuable extract. The best performance was achieved with tips pruned to a length of 4 mm, as shown in Figure 2B. With this size opening, the volume of 100 µL, which is required for the lateral flow assay to function properly, elute in three large drops that can be visually observed, even if the entire set-up is encapsulated in a transparent polypropylene bag (Figure 3). We do the pruning of the tip with the blade of a carpet cutter, previously treated with disinfectant, while the syringe is in a steel holder (see Supplementary Material), but pruning may also be performed in a simpler, hand-held fashion.

The syringe acting as container is held at a 40° angle to the LFA cassette. There are two positions for the syringe. In extraction mode, the LUER fit opening at the tip of the syringe is at its highest position to prevent premature leaking of the extract. Our preferred procedure involves self-sampling under the supervision of a medically trained member of the team (‘superuser’) undergoing pooled testing. The superuser sets up the device, drips the necessary volume of extraction buffer into the syringe, places the set-up in the transparent bag and supervises the collection of the specimens. Sampling occurs in a fume hood or well-ventilated area with a minimum distance of 3 m between individual team members and superuser. The members of the team enter their swabs into the container, one by one, wearing an inexpensive polyethylene glove on the hand with which they handle the swab, and the superuser, wearing medical-grade gloves and a surgical mask or FFP2 mask, then closes the bag and sprays it down with disinfectant from the outside. After a brief interval to allow for evaporation of the alcohol-based disinfectant, the superuser performs the extraction, elution and analysis while the entire set-up is inside the closed bag (Figure 3).

Figure 3A shows how the extraction set-up is inserted into the sealable, transparent polyethylene bag of sufficient mechanical stability and optical transparency. Mixing is achieved by rotating and laterally moving the swabs immersed in the buffer for 3 min. This step requires some dexterity and experience to ensure that the extract is as homogeneous as possible. It is easier to perform this step with more liquid, but dilution lowers the concentration of the antigen and is therefore undesirable. After mixing, the syringe is switched into elution mode by rotating it 180°, so that the LUER fit is in its lowest position, allowing the extract to drip into the specimen well of the cassette. To ensure that the necessary volume is liberated, the swabs should be slightly moved up to avoid blockage of the exit port, and the swabs should be gently squeezed against the wall of the syringe. Again, this calls for dexterity and may be rehearsed without potentially infectious material first by operators new to the extraction device. It is also critical to perform the elution with close visual inspection to ensure that the necessary number of drops is applied.

**Extraction and Analytical Assay.** Pooling is expected to increase the total volume of the extract. To minimize the resulting loss in signal, the concentration of the antigen should be kept as high as possible. To achieve this, the analytical procedure was optimized. First, we gravimetrically determined the volume absorbed by swabs upon immersion in water for 5 s, with the aid of an analytical balance. We found that the NFS-1 flocked style nylon swabs provided with the Abbott test kit absorb 56.7 ± 3.0 µL under those conditions. Given the uncertainty of the viscosity of the real specimen, we refer to these swabs as ‘50 µL swabs’, assuming that they will take up approximately 50 µL specimen. The less expensive 5 mm cotton wool swabs with wooden stem take up 101.3 ± 4.1 µL of water under the same conditions, and we refer to those swabs as ‘100 µL swabs’.

We assumed that thorough mixing after pooling of the swabs produces a homogeneous liquid as extract. The total volume of this extract will be the sum of the volume of the extraction buffer employed and the volume entered with the specimens. Again, for simplicity, we assumed that the latter volume is either 50 µL or 100 µL per person participating in the pooled test. The standard protocol, as described in the user manual for the Abbott Panbio COVID-19 Ag Rapid Test, calls for 300 µL extraction buffer. The bottle provided with the test kit (25 tests) contains 9 mL or 360 µL per test. Assuming that leaving no more than a small residual volume in the buffer bottle is not critical, we used up to 350 µL extraction buffer per pooled test. We determined that this equals 13 drops from the Abbott bottle. Unless otherwise noted, one positive control swab was included in all laboratory assays as a constant source of antigen to be detected.

**Figure 4:**
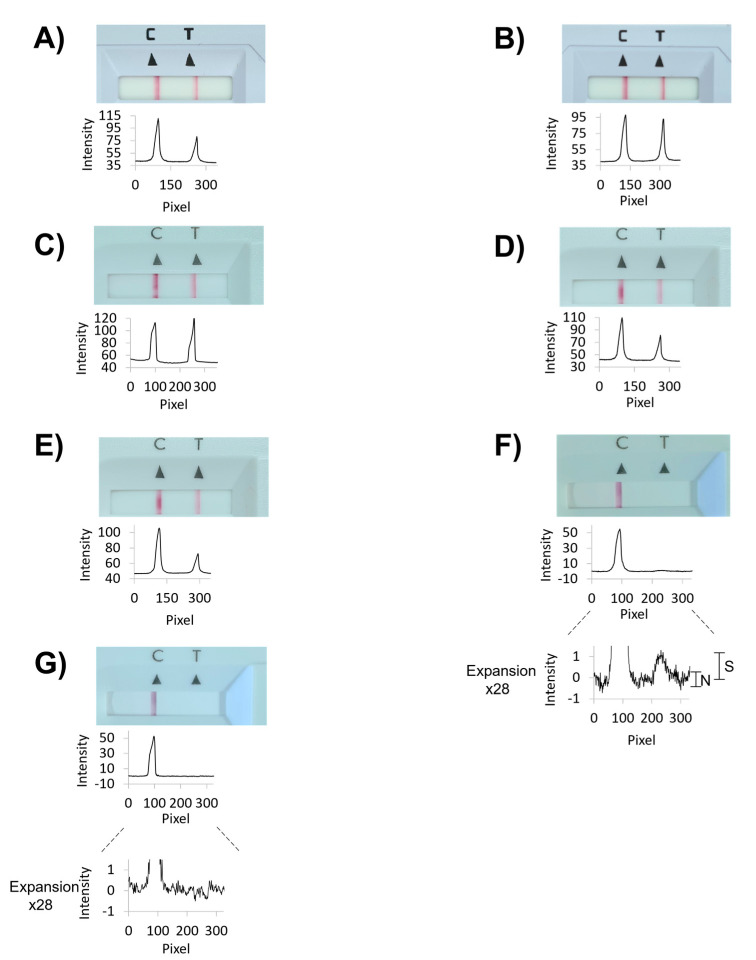
Representative results from rapid antigen tests with or without pooling. Photographs of the read-out window of lateral flow assay cassettes are shown, together with signal intensity plots for each test results, as obtained by integration with ImageJ. Except when otherwise noted, photographs were taken 15 min after the start of the assay using the Abbott PanBio test. (**A**) Standard Roche test with a single positive control swab. (**B**) Same as (**A**) but after 30 min. (**C**) Abbott assay with a positive control swab only. (**D**) Pool of five nasopharyngeal 50 µL swabs from healthy volunteers and one positive control swab. (**E**) Five cotton wool 100 µL swabs and one positive control swab. (**F**) Same as (**E**), but with 32-fold dilution with extraction buffer. An expanded view of the integration plot is shown below the original plot. (**G**) Six pooled cotton wool swabs from healthy volunteers. An expanded view of the integration plot is shown below the original plot. See Table 1 for numerical values obtained by integration and further experimental details.

Figure 4 shows results from laboratory assays performed with our device, and Table 1 lists the corresponding data numerically. The numerical values for signal intensities of the test (SARS-CoV-2 antigen) and control lines were obtained by analyzing photographs taken with the camera of a smartphone with the free software ImageJ, available from the National Institutes of Health, as detailed in the Supplementary Information. The plots below each photograph of the read-out window of the test cassettes in Figure 4 are the results of the integration of the gray value data calculated with ImageJ.

For the conventional, single-swab assay, run with the positive control swab alone, as recommended in the user manual of the Panbio Abbott test, the intensity of the test line was 86% of that found for the control line after 15 min assay time (Figure 4A and entry 1 of Table 1). Next, five 50 µL swabs with specimens from healthy volunteers among the authors plus the dry positive control swab were extracted with 350 µL of the extraction buffer (600 µL total volume, Vtot), a signal corresponding to 55% of that for the individual test was measured (entry 2 of Table 1). This is the expected value, based on the approximately two-fold dilution.

**Figure 5:**
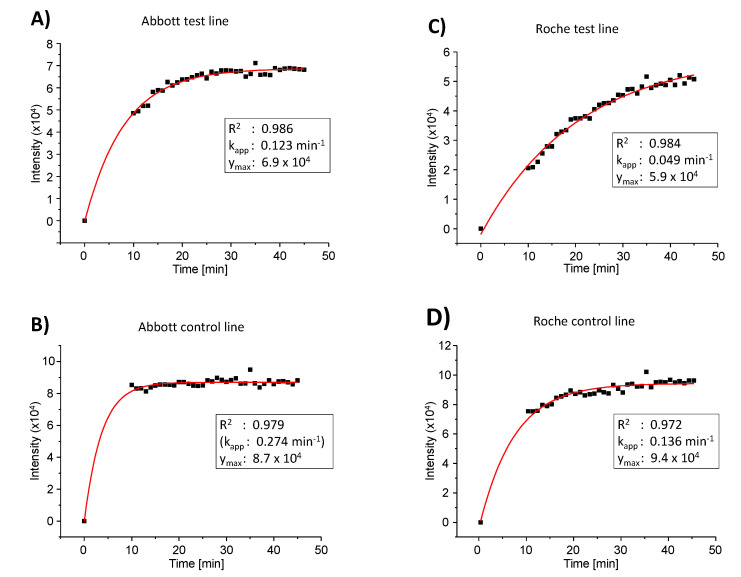
Kinetics of binding of colloids at the test and control lines of the PanBio test from Abbott, or the Rapid Antigen test from Roche Diagnostics, as determined by image analysis of photographs taken at stated intervals after applying the antigen-containing extract of the Abbott test to the test cassettes. Five specimens of healthy and negatively tested volunteers among the authors and one positive control swab (Abbott) were extracted with the buffer of the Abbott test in the device of Figure 1, Figure 2 and Figure 3. The extracts were then applied to each of the test cassettes. Data collection started 10 min after the extracts were applied. Black boxes are experimental data points and red lines are fit to an exponential kinetic model using the fit equation y = y_max_ · (1 − exp(k_app_ · t)), where y are the intensity values, t is the time of data acquisition, y_max_ is calculated maximum intensity at infinite time, and k_app_ is the apparent rate constant for the process. Boxes below each plot give the numerical values obtained. The apparent rate constant for the Abbott control line is not defined by a sufficient number of early data points and is therefore reported in parentheses. (**A**) Abbott test line, (**B**) Abbott control line, (**C**) Roche test line, and (**D**) Roche control line. Note the different scales of the y-axes for the different plots.

We then performed an individual test with the Roche test, and found less signal (entry 3 of Table 1). Visual inspection indicated an increase in intensity for the test line of the Roche test when allowed to develop longer than 15 min. This prompted us to collect a series of images over time in order to obtain the kinetics of colloid binding to the test line. The results for the Abbott and the Roche test are shown in Figure 5. The signal for the SARS-CoV-2 antigen did indeed rise more slowly for the latter assay, and even after 30 min (the upper time limit for read-out specified by the manufacturer), the value was below that for the PanBio test (entry 4, Table 1). Since rapid testing is desirable, we decided to focus on the Abbott test in all subsequent work.

We next studied how dilution affects the signal in the lateral flow test. For this, we switched to the 100 µL swabs, which have a larger capacity and thus the potential to compensate for dilution. Only the positive control swab was left unchanged, as no larger volume version of this was available to us. In the first set of experiments, we used wet 100 µL swabs, pre-hydrated with sterile water and an extraction buffer diluted two-fold with the same water. This gave 37% of the control signal, i.e., considerably more than the 27% expected based on the estimated dilution factor but was not pursued further over concerns that the dilution may change the characteristics of the extraction medium [10] and that the extra cost (and effort) to provide individual sterile vials with dilution water to each participant would become prohibitive. Still, it is an interesting finding, as it suggests stronger binding of the antigen-loaded gold nanoparticles to the surface-immobilized antibodies in medium with lower ionic strength than in standard medium. Our previous studies with DNA-coated gold nanoparticles on other surfaces [18,19] gave the opposite effect when lowering the salt concentration, which may be due to the different biopolymers involved and characteristics of the corresponding molecular recognition events. There was also the possibility that the signal may not increase linearly with concentration due to multivalency effects, [20] which motivated dilution experiments (vide infra).

In experiments performed with dry 100 µL swabs, we noted that incomplete hydration during sample collection occasionally led to difficulties in obtaining sufficient extract to apply to the test cassette. Therefore, we adopted a procedure starting with wetting the swabs with saliva for 30 s, followed by oro- and nasopharyngeal sampling. The saliva makes nasopharyngeal sampling less painful and avoids that partially wetted swabs take up too much of the buffer solution. The combined oro- and nasopharyngeal sampling is similar to what is typically done with smaller, dry swabs when collecting specimen for PCR tests. Our procedure that involves saliva plus specimen collected by swabbing is in agreement with the recommendations of a study with 659 patients that employed the Panbio test and that showed high sensitivity for the combination of saliva and nasal sampling [21]. The ability to detect infections with saliva was also confirmed by others [22,23]. Using this approach for sampling, combined with the 350 µL of extraction buffer available from the test kit, the 100 µL extract necessary for running the lateral flow assay was obtained without the need for any additional fluid. We considered this the optimal procedure under our conditions and opted for six-fold pooling with 100 µL swabs as our preferred mode of performing pooled tests.

When the optimized team test was performed eight times with samples from volunteers among the authors, we obtained a signal intensity of 43 ± 6% (mean ± one SD) of that for the single swab standard procedure (entry No. 6, Table 1). A negative control swab or six specimen-holding swabs from healthy, negatively tested volunteers gave no detectable signal (entry 7 of Table 1). We then performed exploratory experiments to determine the limit of detection. For this, the extract from an assay performed as for entry 6 of Table 1, was diluted with extraction buffer in 1:2 dilution steps, resulting in extracts with 2-, 4-, 8-, 16-, and 32-fold lower antigen concentration (entries 8–12, Table 1). Even for the most dilute extract, a positive result was detectable when using image analysis after photography, with a signal-to-noise ratio of approximately 2:1 (Figure 4F). Zooming in on the baseline of the negative control assay did not show maxima of similar intensity (Figure 4G).

A quantitative analysis of the results of the kinetics study with a monoexponential model, using the software Origin Pro, version 8.0, gave the numerical data presented in the boxes of Figure 5. Except for the control line of the Abbott test, where almost full signal intensity had been reached at the starting point of data collection, satisfactory fits were obtained with this simplified model of colloid binding (R^2^ values > 0.97). The apparent rate constant of binding (k_app_) was found to be approximately 2.5-fold larger for the Abbott test than for the Roche test. The absolute value of the maximum signal intensity was found to be 1.17-fold higher for the Abbott test versus the Roche test. The calculated relative signal intensity (test versus control line) at infinite time was determined as 0.79 for the Abbott test and 0.63 for the Roche test, confirming the conclusions from the initial qualitative analysis mentioned above.

**Figure 6:**
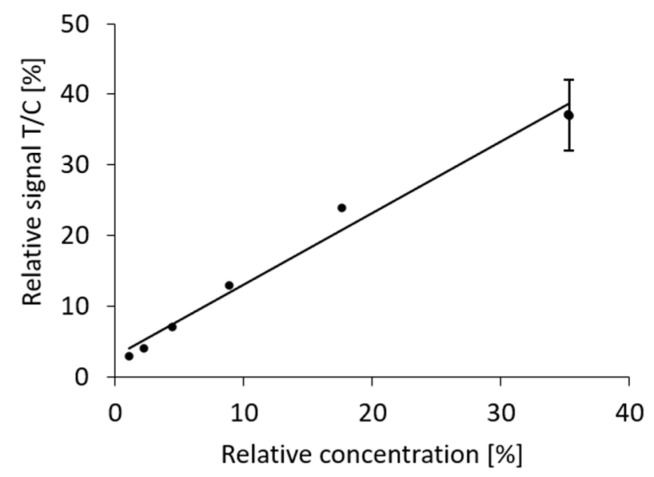
Correlation between antigen concentration after pooling and dilution and the relative signal (intensities test line/control line) observed with PanBio test cassettes. The data was obtained by integration of the signals measured in the dilution series of Table 1 (entries 6 and 8–12).

We then asked whether the effect of dilution is predictable. For this, we decided to plot the relative signal (test versus control strip) from the dilution series against the relative concentration of the antigen. The relative concentration was calculated from the total volume of the initial extract and subsequent dilution steps and expressed as a percentage of the concentration in the standard extract of the positive control swab. A linear correlation with an R^2^ value of 0.98 was found (Figure 6).

**Figure 7:**
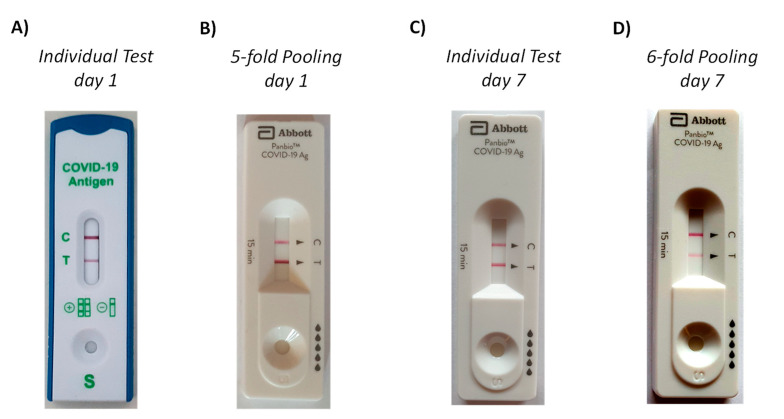
Photographs from case study involving a patient with PCR-confirmed COVID-19: images of test cassettes after individual or pooled tests with one swab from the patient and four or five swabs from a healthy volunteer among the authors at the time point after onset of symptoms given. (**A**) Individual test with Lyher Covid-19 Antigen Rapid test at noon on the day of onset of symptoms, (**B**) pooled assay with Abbott test five hours after the individual test, (**C**) individual test with Panbio Abbott system after one week, (**D**) six-fold pooling test after one week, immediately after the individual test.

A full clinical validation was beyond the scope of our study. However, a SARS-CoV-2 infection among the authors (unrelated to our experiments) provided the data for a case study. Upon onset of symptoms, a commercial rapid antigen test (Lyher Covid-19 Antigen Rapid test) was performed that gave a positive result (Figure 7A). While the symptoms were increasing, a test with 5-fold pooling (four from a healthy volunteer among the authors, one from the patient) was then run approximately 5 h after the initial test. This test employed the Abbott PanBio cassette and our extraction device and gave a strong positive signal, despite the pooling (Figure 7B). The patient underwent PCR testing with NSP swab collection of specimens by health care professionals on the subsequent day, confirming the COVID-19 diagnosis. Seven days after the onset of symptoms and the first rapid antigen tests, a second set of tests was run. This time, both the individual and the pooled test employed the Abbott Panbio system. Both specimens were taken from the same nostril, with the first swab for the individual test being applied immediately prior to that for the pooled test, which employed five swabs from the same healthy volunteer as in the 5-fold pooling assay run earlier. As expected, the individual test (Figure 7C) gave a stronger signal than the pooled test (Figure 7D), and a clear positive signal was obtained in either case. The patient was free of symptoms after ten days, and an individual test run at this time point with another commercial test gave little signal. All photographs were taken at the point of care, without an attempt to control the illumination of the test cassettes, unlike the laboratory experiments described above. As expected for different tests systems and regimes of this ad hoc validation, there are quantitative differences between individual and pooled tests. Importantly, unambiguously positive signals were obtained in either of the tests with swab pooling.

Finally, we performed a pilot project in a laboratory course at the University of Stuttgart. For this, groups of five or six persons were assigned, including students (master program) and teaching assistants and/or faculty members. Written consent was obtained from all participants, and each team performed two tests per week for the duration of the three-week course. Each participant was issued one swab on the day of testing. All tests used the settings of entry 6 of Table 1. The results are compiled in chapter 4 of the Supplementary Material. No false positive results were obtained, and the protocol was deemed suitable for practical courses at institutions of higher learning by the participants.

## 4. Discussion

Our data indicate that swab pooling in a device is a valid option for routine monitoring in settings where the available resources limit the frequent use of individual lateral flow tests. We note that the results of Table 1 should be regarded as the lower limit of the signal we expect for a positive specimen in real life monitoring set-ups. The positive control swab from Abbott is designed for an assay with 50 µL swabs, but 100 µL swabs are employed in our preferred tests with swab pooling. So, while the total volume has gone up approximately three-fold with six-fold pooling, the amount of specimen is increasing approximately two-fold over that of the standard swab, resulting in a numerical decrease in concentration of antigens by a factor of 1.5 only.

In our current testing regime, the operators of the team test obtained a 90 min in-person training by a health care professional on the use of rapid antigen tests (Roche). In house-training on the use of our device for pooling and analysis, as well as data analysis, took another 60 min. Instructing the team members without operator status and obtaining informed consent required approximately 30 min, so that the overall time effort is limited. The recommended conditions for testing (temperature, humidity etc.) are not different from those recommended by the manufacturer of the test cassette and should be obtained from the manuals of respective tests.

Even though only fluids intended by the original procedure are being used, a validation of the pooling in a full clinical study or field study is required. Assuming that this validation will confirm the results of our exploratory experiments, a ‘team test’ approach may facilitate surveillance for viral infections at approximately five-fold reduced costs at the six-fold pooling level. In an in-house validation, we experience better compliance with a twice weekly voluntary test regime when testing was performed as a team test than with individual tests requiring medical appointments. For ordinary team members, the time effort is well below 5 min per test, and the effort of the ‘superuser’ is approximately 30 min per team test, 15 min of which is waiting time for the test to develop. The ability to run the tests at the workplace with low set-up costs and minimized infection risk due to encapsulation, as well as the self-sampling option, contribute to the attractiveness of the pooling approach. Follow-up in positive cases should be done with more sensitive methods, such as RT-PCR, and negative results should not be the basis for medical decisions or a reduction or protective measures, such as mask wearing. In our laboratories, we agreed on the following measures. When a team test gives a positive result, all members of the team leave the premises and self-isolate until they have undergone individual tests at an official testing site or clinic. The encapsulated test set-up, including the holder, are thoroughly disinfected from the outside and disposed of as mandated by local regulations for infectious material.

The goal of monitoring is to detect infectious individuals early, using a process that is sustainable, both financially and in terms of the time effort involved. The manual of the Panbio test states that it has a detection limit of 2.5 × 101.8 TCID50 of SARS-CoV-2 and a sensitivity of 94.1% ‘with samples of Ct values ≤ 33’. Data from an early study [7], as well as data from a more recent field study [24], indicate that the Abbott Panbio rapid antigen test reliably detects ≥ 90% of individuals with a viral load corresponding to a Ct value of ≥ 28. A multicenter study found a sensitivity of 95.8% for Ct values below 25 when testing within the first 7 days after onset of symptoms [25]. A viral load of Ct > 28 is at the upper limit of what is believed to make a person infectious [7,9,26,27]. Therefore, we have reason to believe that the approximately two-fold drop in sensitivity observed for six-fold pooling keeps the detection limit within the range that successfully identifies active spreaders of the SARS-CoV-2 virus, particularly when combined with the gain in sensitivity that can be achieved with image analysis. We expect the uncertainty associated with sampling by oro- and nasopharyngeal swab probing to be reduced when including saliva, as in our preferred procedure. The uncertainty may be reduced further with improvements over standard swabs [28] or extraction buffer composition, as discussed for tests detecting nucleic acids [29]. We note that our procedure deviates from that recommended by Abbott and that a clinical study is planned to obtain more robust data from outbreaks of COVID-19. With such data, it will be easier to provide more safety to families, work teams, musicians, and other groups threatened with the spread of viral infections through frequent testing with swab pooling.

## 5. Conclusions

With the pooling strategy described here, a roughly five-fold reduction in material cost may be achieved, while the accompanying reduction in sensitivity is only approximately two-fold, depending on the type of swabs employed. This reduction in sensitivity corresponds to approximately one Ct number (or ‘average ct’) [24] and may be compensated by increasing the sensitivity via quantitative image analysis, using a smartphone and free image analysis software. In the absence of a digital camera, only operators with unimpaired vision should read out the test result. Even without compensating measures, a reduction by only one Ct number should not outweigh the gain in practicability, which results from a simple, rapid, point-of-need procedure that does not require medical personnel or laboratory infrastructure. Pooling not only reduces the cost, but also allows the testing of more people when the availability of LFA test cassettes is limiting. Our approach minimizes the risk of infection when supervised self-sampling at a safe distance is practiced, and when the analytical procedure is performed in a sealed bag. Independent of the details of the protocol, the inexpensive device described here may help to reduce the transmission of SARS-CoV-2 and other viruses that threaten the livelihood of many communities. Our method should be validated further.

## Figures and Tables

**Table 1:** Results of rapid antigen test with or without pooling of swabs.

Entry No.	Manufacturer	No. of Swabs ^(a)^	Type of Swabs Used [µL Capacity] ^(b)^	Estimated V_tot_ [µL] Incl. Specimen	Dilution with Additional Liquid	Relative Intensity Test/Control [%]	Signal Relative to Simplex Assay ^(c)^ [%]
1	Roche	1	50	350	-	53	-
2 ^(d)^	Roche	1	50	350	-	75	-
3	Abbott	1	50	300	-	86	100
4	Abbott	6	50	600	-	47	55
5	Abbott	6	100	1100 ^(e)^	wet swabs ^(f)^	32	37
6	Abbott	6	100	850	-	37 ± 5 ^(g)^	43 ± 6 ^(g)^
7	Abbott	6 ^(h)^	100	950	-	0	-
8	Abbott	6	100	850	2-fold ^(i)^	24	28
9	Abbott	6	100	850	4-fold ^(i)^	13	15
10	Abbott	6	100	850	8-fold ^(i)^	7	8
11	Abbott	6	100	850	16-fold ^(i)^	4	5
12	Abbott	6	100	850	32-fold ^(i)^	3	4

^(a)^ Unless otherwise noted, one of the swabs was a positive control swab (Abbott). ^(b)^ Positive control swab was added in dry form. ^(c)^ Simplex assay is an assay with a single swab, according to the manufacturer’s manual of the Abbott LFA test (related to entry No. 1). ^(d)^ Same experiment as for entry No. 1 but after 30 min. ^(e)^ 300 µL extraction buffer plus 300 µL sterile water. ^(f)^ Pre-hydrated with sterile water prior to sampling. ^(g)^ Mean ± one standard deviation from eight experiments. ^(h)^ No positive control swab included. ^(i)^ Extract prepared as for entry No. 6, but then diluted with extraction buffer.

## Supplementary Materials

The following is available online at https://www.mdpi.com/article/10.3390/diagnostics11071290/s1: the protocols for image analysis, data plotting and the pruning of syringes, as well as the results of the pilot project in the laboratory course.
