# Supplementary material for: The Effect of Pooling on the Detection of the Nucleocapsid Protein of SARS-CoV-2 with Rapid Antigen Tests
Source: Diagnostics (Basel). 2021 Jul 19;11(7):1290. doi: 10.3390/diagnostics11071290 (PMC8303537; doi:10.3390/diagnostics11071290)

Supplementary Information

for manuscript entitled

The Effect of Pooling on the Detection of the Nucleocapsid Protein of SARS-CoV-2 with Rapid Antigen Tests

by

Tim Berking, Sabrina G. Lorenz, Alexander Ulrich, Joachim Greiner, Jennifer Bremer, Christina Wege, Tatjana Kleinow and Clemens Richert.

Contents

1. Protocol for Image Analysis
2. Protocol for Data Plots
3. Process of pruning syringes
4. Results of the pilot project in a laboratory course at the University of Stuttgart

1. Image Analysis

Pictures of test cassettes for the numerical analysis of signal intensities were taken with a Samsung Galaxy S20+ (SM-G985F) smartphone in photo mode, using the wide angle option, without a flashlight, in 3:4 format. The test cassettes were photographed in daylight/fluorescent bulb laboratory light with an attempt to achieve even light exposure on the lab bench where pictures were taken.

We also tested the pro mode (ISO 100, 1/90s shutter speed, auto focus, 4000-5000 K white balance) for raw picture data, and compared them to the data in photo mode to verify that the picture is not falsified by the AI of the smartphone used. Both data showed no discernable difference in relative signal intensity.

The pictures were transferred to a laptop *via* USB cable and opened in ImageJ (downloaded from the ImageJ homepage). In subsequent steps, the pictures were turned into a 8-bit type and inverted, as documented below.

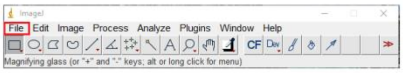

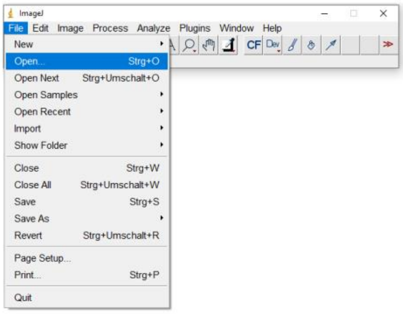

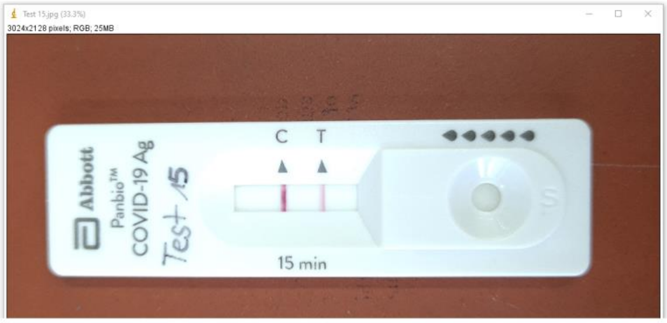

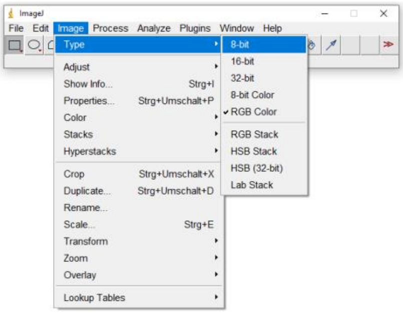

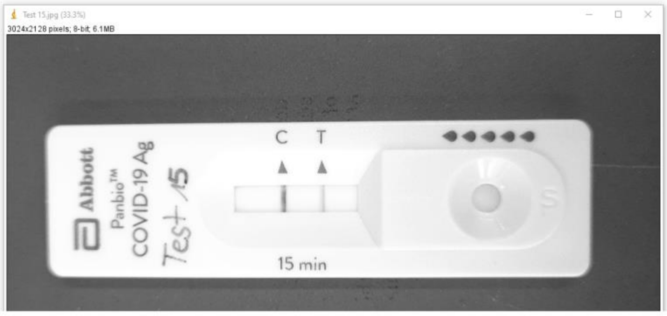

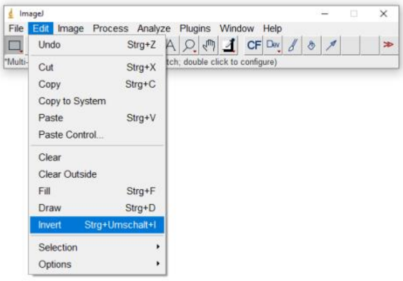

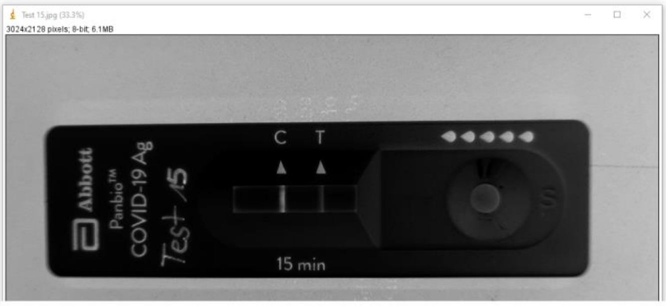

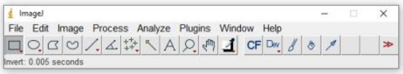

Rectangular areas of the identical size were chosen and marked around the control line, background (area between control line and test line) and the test line using “rectangle”. The gray value intensities were measured (Tab “Analyze” → Set Measurements), adjusted by activating "Integrated density" and automatically listed in the table “Results” as “IntDen”, as documented graphically below.

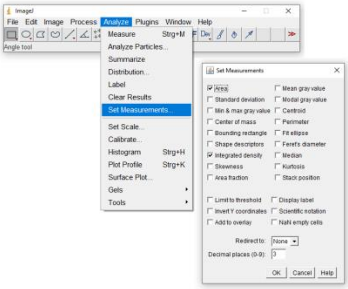

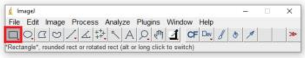

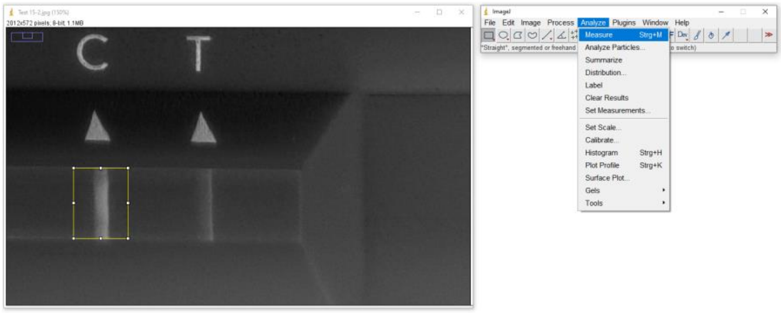

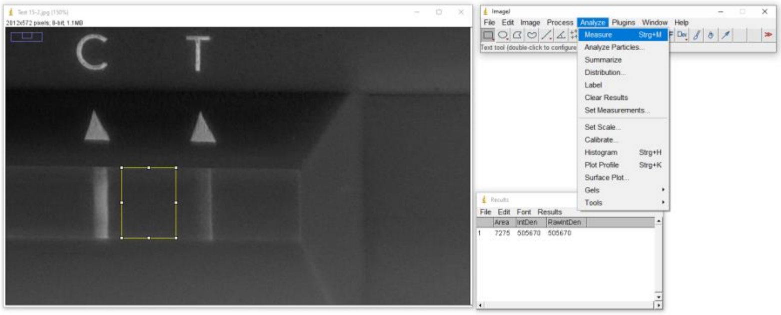

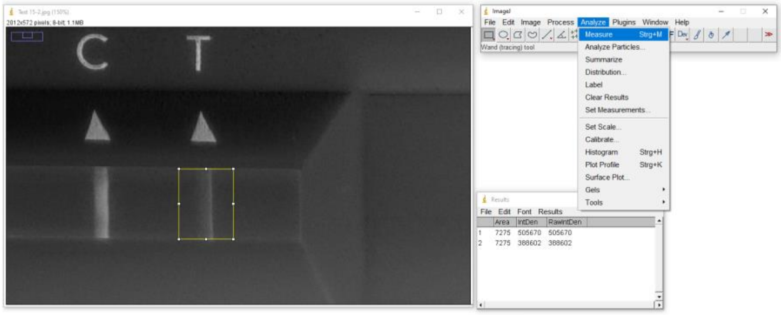

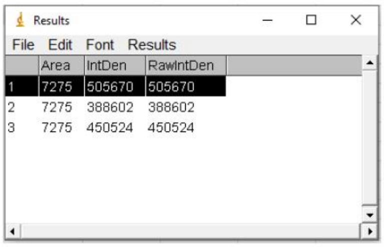

	Area	IntDen	RawIntDen
1	7275	505670	505670
2	7275	388602	388602
3	7275	450524	450524

To calculate the relative signal intensity of test line versus control line, equation 1 was used:

$$Intensity\ test\ relative\ to\ control\ [\%] = \frac{intensity\ test\ line - intensity\ background}{intensity\ control\ line - intensity\ background} * 100 \qquad (1)$$

Furthermore, we used equation 2 to calculate the intensity of the test result of our pooling tests in relation to the simplex assay involving standard extraction of a single positive control swab (Abbott):

$$Intensity\ test\ related\ to\ simplex\ assay\ [\%] = \frac{Intensity\ test\ relative\ to\ control\ [\%]}{intensity\ test\ relative\ to\ control\ of\ simplex\ assay} \qquad (2)$$

2. Protocol for Data Plots

In order to graphically display the signal intensities of the control and test line, the area of the test strip was marked using a rectangle and the plot was made using “Analyze” → “Plot Profile”. The obtained plot was then displayed numerically with “List” and saved as an Excel file.

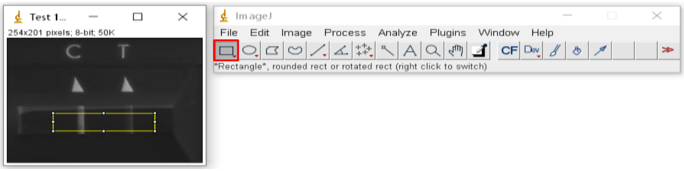

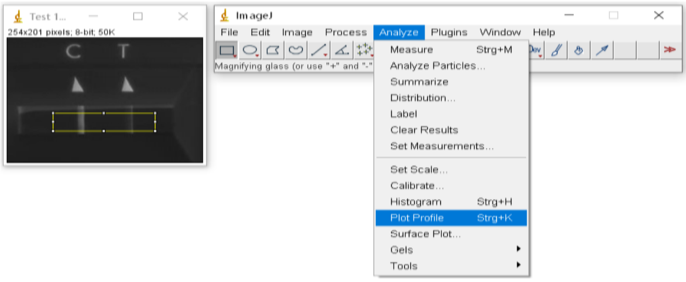

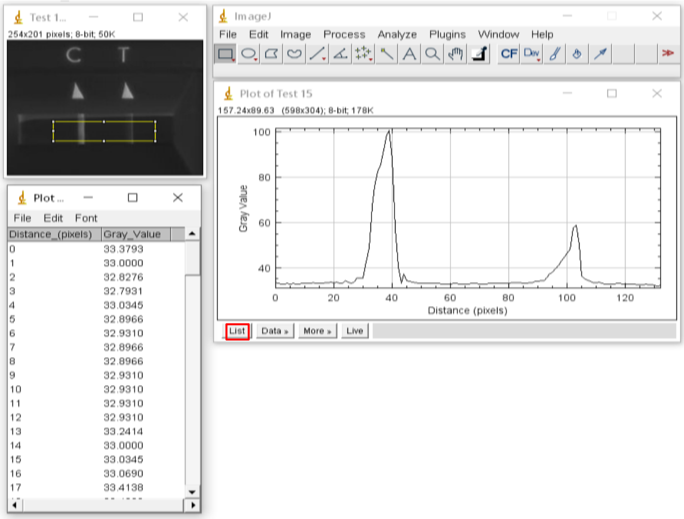

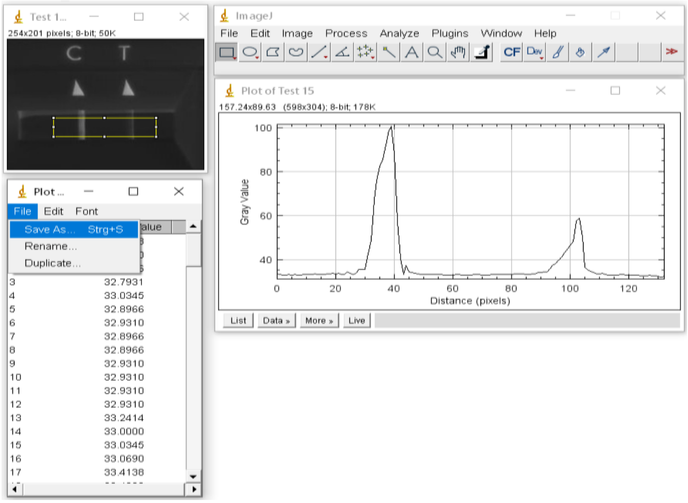

3. Pruning Syringes

The LUER outlet of the syringe should be shortened to avoid a loss of extract volume that otherwise remains in the opening.

Components employed in the procedure

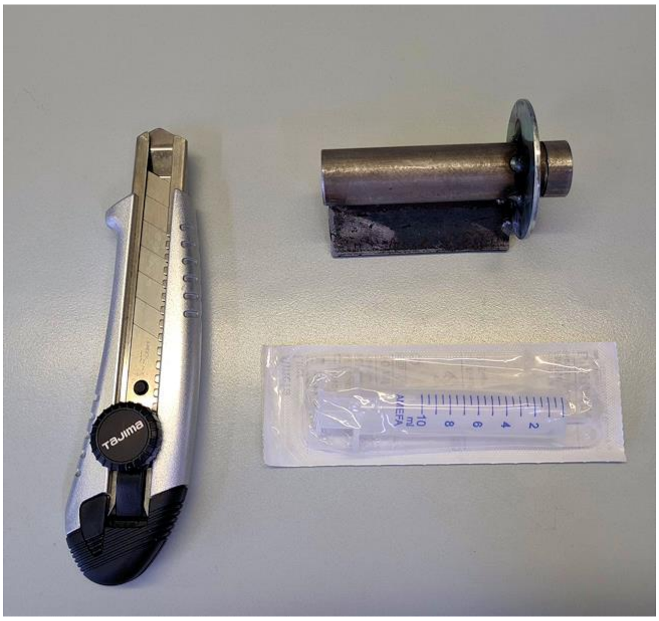

Pruning the tip of the syringe in the holder

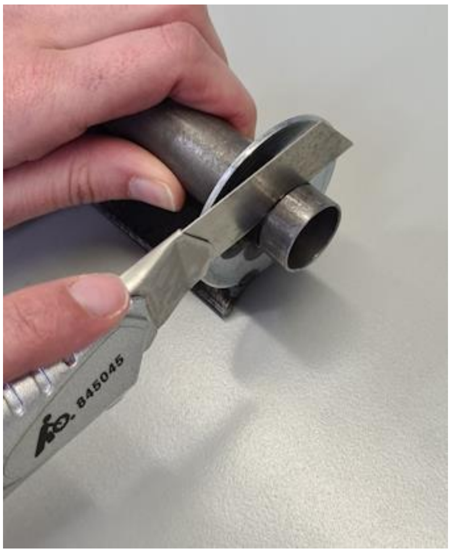

4. Results of the pilot project in a laboratory course at the University of Stuttgart

Shown are the test schedule, photographs of the test cassettes and image analysis plots.

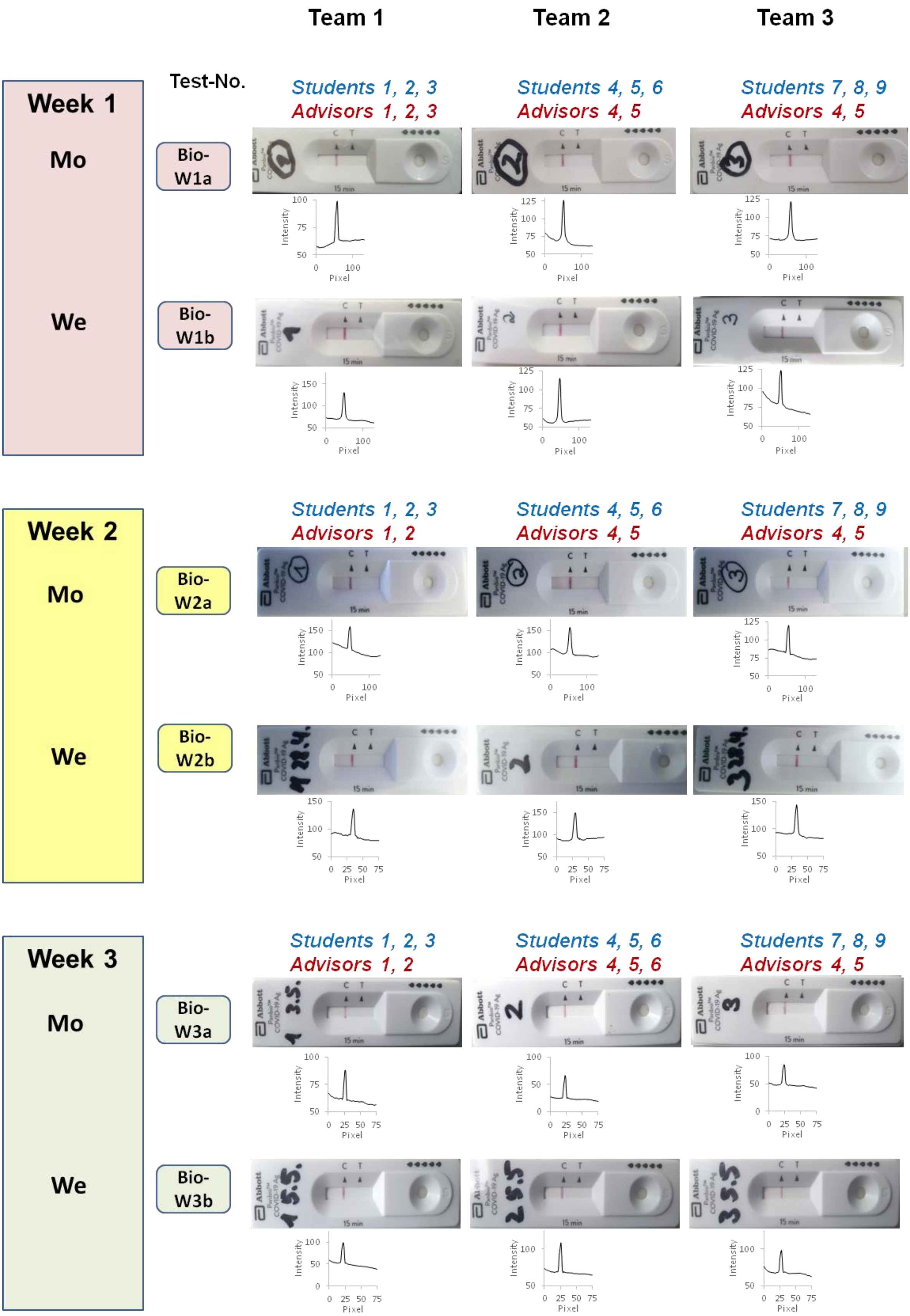

Supplement: Supplementary file 1 [file diagnostics-11-01290-s001.zip › diagnostics-1295641-supplementary.pdf]
